# Edge‐Sharing Octahedrally Coordinated Ni—Fe Dual Active Sites on ZnFe_2_O_4_ for Photoelectrochemical Water Oxidation

**DOI:** 10.1002/advs.202301869

**Published:** 2023-06-01

**Authors:** Zhiyong Jiang, Xiaodi Zhu, Zhiyu Wang, Wei Liu, Wensheng Yan, Kevin Sivula, Jun Bao

**Affiliations:** ^1^ National Synchrotron Radiation Laboratory University of Science and Technology of China Hefei Anhui 230029 China; ^2^ State Key Laboratory of Fine Chemicals School of Chemical Engineering Dalian University of Technology Dalian 116024 China; ^3^ Laboratory for Molecular Engineering of Optoelectronic Nanomaterials (LIMNO) École Polytechnique Fédérale de Lausanne Station 6 Lausanne 1015 Switzerland; ^4^ Key Laboratory of Precision and Intelligent Chemistry University of Science and Technology of China Hefei Anhui 230026 China; ^5^ iChEM (Collaborative Innovation Center of Chemistry for Energy Materials) Hefei Anhui 230029 China

**Keywords:** dual active sites, molten salt, spinel ferrites, ultra‐low onset potential, water splitting

## Abstract

The structural properties of octahedral sites (B_Oh_) in spinel oxides (AB_2_O_4_) play vital roles in the electrochemical performance of oxygen‐related reactions. However, the precise manipulation of AB_2_O_4_ remains challenging due to the complexity of their crystal structure. Here, a simple and versatile molten‐salt‐mediated strategy is reported to introduce Ni^2+^ in B_oh_ sites intentionally on the surface of zinc ferrite (ZnFe_2_O_4_, ZFO) to promote the active sites for photoelectrochemical (PEC) water splitting. The as‐created photoanode (ZFO‐MSNi) shows a remarkable cathodic shift of ≈ 450 mV (turn‐on voltage of ≈ 0.6 V_RHE_) as well as three times the 1‐sun photocurrent density at 1.23 V_RHE_ for PEC water oxidation in comparison with bare ZFO. A comprehensive structural characterization clearly reveals the local structure of the introduced Ni^2+^ in ZFO‐MSNi. Fewer surface trapping states are observed while the precisely introduced Ni^2+^ and associated neighboring Fe^(3‐*σ*)+^ (0<*σ*<1) sites unite in an edge‐sharing octahedral configuration to function as Ni—Fe dual active sites for PEC water oxidation. Moreover, open circuit potential measurements and rapid‐scan voltammetry investigation give further insight into the enhanced PEC performance. Overall, this work displays a versatile strategy to regulate the surface active sites of photoelectrodes for increasing performance in PEC solar energy conversion systems.

## Graphical abstract

Edge‐sharing octahedrally coordinated Ni^2+^—Fe^(3‐*σ*)+^ (0<*σ*<1) dual active sites are precisely constructed on the surface of ZnFe_2_O_4_ photoanodes with fewer trapping states through a simple molten‐salt‐mediated strategy. A remarkable cathodic shift of ≈ 450 mV with photocurrent onset potential as well as obviously enhanced 1‐sun photocurrent density is achieved for photoelectrochemical water oxidation.

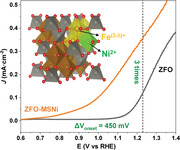

## 1 Introduction

Photoelectrochemical (PEC) “artificial leaf” technology, which employs photoanodes and photocathodes to store renewable solar energy in the form of chemical bonds (e.g., H_2_ from water splitting) is one of the most promising energy conversion technology to realize scalable and cost‐effective solar fuel production.^[^ ^1^, ^2^, ^3^ ^]^ In this regard, photoelectrode materials hold great importance for the conversion efficiency and the economic performance of PEC devices. Despite some well‐exploited metal oxides (e.g., Fe_2_O_3_,^[^ ^4^, ^5^ ^]^ BiVO_4_,^[^ ^6^, ^7^ ^]^ and WO_3_^[^ ^8^, ^9^ ^]^), spinel ferrites (MFe_2_O_4_, M represents divalent metal ions, such as Zn^2+^, Mg^2+^, Cu^2+^, Ni^2+^, etc.) show great potential as photoelectrodes for PEC water splitting by converting solar energy into hydrogen owing to its suitable band structure, excellent visible light capture ability, photochemical stability as well as flexible structural adjustments.^[^ ^10^, ^11^, ^12^ ^]^ However, the performance of state‐of‐the‐art MFe_2_O_4_‐based photoelectrodes is still far below the values required for practical hydrogen production. Thus, developing effective strategies to promote their PEC performance is urgent yet challenging.

As a subgroup of AB_2_O_4_ of which tetrahedrally coordinated (A_Td_) and octahedrally coordinated (B_Oh_) sites can be accommodated by the same or different transition metals, MFe_2_O_4_ consists of M^2+^ cations and Fe^3+^ cations with certain distribution at A_Td_ sites and B_Oh_ sites in oxygen cubic close‐packed crystal structure.^[^ ^11^, ^13^, ^14^, ^15^ ^]^ Particularly, the structural properties of B_Oh_ cations in the spinel framework play important roles in the electrochemical performance of oxygen‐related reactions, which even can serve as a descriptor.^[^ ^16^, ^17^ ^]^ Since AB_2_O_4_ possesses a configurable structure with an internal A_Td_—O—B_Oh_ corner‐sharing and B_Oh_—O—B_Oh_ edge‐sharing geometry, it can be deduced that the neighboring cations at both A_Td_ sites and B_Oh_ sites are capable of regulating the electronic structure of B_Oh_.^16^, ^18^ ^]^ Furthermore, the regulatory effect of the neighboring cations at B_Oh_ sites is more significant than that of those at A_Td_ sites. Thus, manipulating the cations types at B_oh_ sites in MFe_2_O_4_ can become an effective approach to promote their PEC performance.

In this work, we report a method to introduce Ni^2+^ in B_oh_ sites intentionally and precisely on zinc ferrite (ZnFe_2_O_4_, ZFO) surface as a proof of concept to promote the active sites for PEC water oxidation through a simple and versatile molten‐salt‐mediated strategy (the as‐obtained photoanode is labeled as ZFO‐MSNi). The thorough X‐ray absorption spectroscopy and PEC investigation clearly show the importance of the edge‐sharing octahedral configuration of Ni^2+^—Fe^(3‐*σ*)+^ (0<*σ*<1) dual active sites compared to the pristine single Fe^3+^ sites. Besides, the fewer surface trapping states on ZFO‐MSNi benefiting from the molten‐salts‐mediated treatment also contributes to the improved carriers’ utilization. As a result, the increased photovoltage and reduced overpotential for PEC water oxidation at ZFO‐MSNi photoanode/electrolyte interface is achieved, bringing about a remarkable cathodic shift of ≈ 450 mV as well as an obviously enhanced 1‐sun photocurrent density.

## 2 Results and Discussion

### 2.1 Synthesis, Morphological, and Structural Characterization

**Figure 1:**
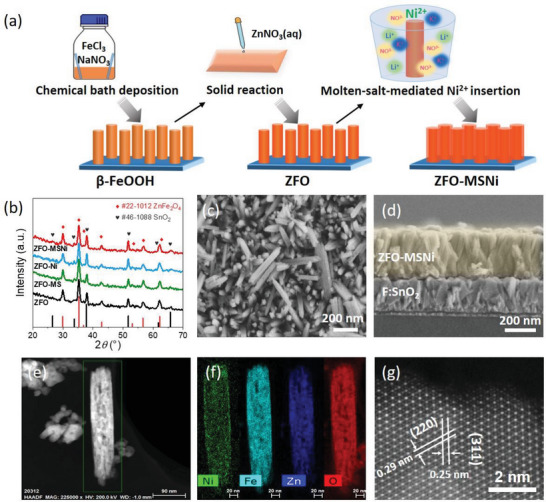
Synthesis, morphology, and structural characterization of ZFO‐MSNi. a) Schematic illustration of the synthesis. b) XRD patterns. c,d) Top‐view and cross‐section SEM images. e,f) HAADF‐STEM image and corresponding EDX mapping. g) The atomic‐resolution HAADF‐STEM image.

As shown in the schematic illustration of the synthesis procedure (**Figure** **1**a), ZFO‐MSNi photoanodes were synthesized by the introduction of Ni species on the pristine ZFO surface through a molten‐salt‐mediated strategy (see the Experimental Section for details). It is worth noting that LiNO_3_/KNO_3_ with eutectic composition was specially selected for our synthetic recipe. The low melting point of eutectic LiNO_3_/KNO_3_ (i.e., 132 °C)^[^ ^19^ ^]^ ensures that this molten‐salt‐mediated treatment only works on the surface region without altering the bulk crystal structure of pristine ZFO. Control samples were also prepared from LiNO_3_/KNO_3_ eutectic molten salt without Ni (ZFO‐MS), and using a conventional Ni impregnation method (ZFO‐Ni). The X‐ray diffraction (XRD) patterns of samples (Figure 1b) verify that ZFO‐MSNi, ZFO‐MS, and ZFO‐Ni all possess the same set of diffraction peaks with the pristine ZFO, which match well with the standard pattern of cubic spinel zinc ferrite (JCPDS #22‐1012). As expected, their crystallinity also exhibits negligible changes, indicating neither the molten‐salt‐mediated introduction of Ni species nor the molten‐salt treatment alone (nor conventional impregnation of Ni species from aqueous precursor) affected the crystal structure of bulk ZFO. The SEM images of samples shown in Figure 1c,d, and Figure S1 (Supporting Information) suggest that the nanorod array‐like morphology (where each nanorod is ≈ 300 nm in length and ≈ 50 nm in diameter) is well preserved after the molten‐salt‐mediated introduction of Ni. The clean surface and the only lattice spacing of 0.29 nm which ascribe to (200) facets of ZFO observed from the TEM and HRTEM images of ZFO‐MSNi nanorods (Figure S2, Supporting Information) exclude the formation of amorphous NiOOH or NiO_x_ species. The aberration‐corrected high‐angle annular dark‐field scanning transmission electron microscopy (AC HAADF‐STEM) image (Figure 1e) and the corresponding energy dispersive X‐ray (EDX) mapping images (Figure 1f) clearly reveal the uniform distribution of the introduced Ni on the surface of the ZFO nanorods. Moreover, the atomic‐resolution HAADF‐STEM image (Figure 1g) shows no lattice distortion, indicating that the introduced Ni species through the molten‐salt‐mediated strategy are probably dispersed on the ZFO surface in the form of single‐atoms.

To further explore the distinct chemical states, electronic structure, spin states, and local coordination environment of the introduced Ni species, comprehensive X‐ray spectroscopy techniques including X‐ray photoelectron spectroscopy (XPS), as well as both soft and hard X‐ray absorption spectroscopy were applied. XPS results (shown in Figure S3, Supporting Information) suggest the successful introduction of Ni^2+^ on the surface of ZFO with a subtle change in the binding energy of the pristine Zn, Fe, and O elements. As listed in Table S1 (Supporting Information), it is worth noting that the surface Ni^2+^ loading of ZFO‐MSNi (≈ 8.5 wt%) was a bit higher than that of ZFO‐Ni (≈ 5.6 wt%). The total loading amounts of Ni species in the whole material were also estimated by inductively coupled plasma atomic emission spectrometry (ICP‐AES) analysis (the results were listed in Table S2, Supporting Information). The Ni loading amount of ZFO‐MSNi (≈ 0.5 wt%) was also higher than that of ZFO‐Ni (≈ 0.4 wt%). These indicate that the molten LiNO_3_/KNO_3_ with eutectic composition is more conducive to introducing Ni^2+^ on the ZFO surface. Additionally, the much less value of the total Ni loading estimated by ICP (≈ 0.5 wt%) compared to that of surface Ni loading evaluated by XPS (≈ 8.5 wt%) also confirmed that the molten‐salt‐mediated Ni introduction dominantly works on the surface region of ZFO.

**Figure 2:**
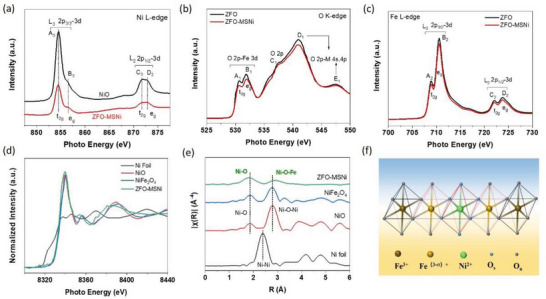
Electronic structure and atomic coordination environment analysis of the introduced Ni^2+^ via molten salt mediated strategy. a) Ni L‐edge XAS spectra of ZFO and ZFO‐MSNi. b) O K‐edge XAS spectra of ZFO and ZFO‐MSNi. c) Fe L‐edge XAS spectra of NiO and ZFO‐MSNi. d) Normalized Ni K‐edge XANES spectra. e) The Fourier‐transformed k^3^‐weighted extended X‐ray absorption fine structure (EXAFS) in R space of ZFO‐MSNi, Ni foil, NiO, and NiFe_2_O_4_ were used as references. f) A schematic diagram of the local structure around Ni^2+^ for ZFO‐MSNi (0<*σ*<1).

Soft X‐ray absorption spectroscopy at the Ni L‐edge (**Figure** **2**a) also reveals the similar valence states and spin states of Ni in ZFO‐MSNi and the reference sample NiO where Ni^2+^ is coordinated with six oxygen in the form of NiO_6_ octahedra. The O K‐edge spectra of ZFO‐MSNi and ZFO are displayed in Figure 2b. In both the pre‐edge region (530–536 eV) and post‐edge region (>536 eV), two samples exhibit similar spectra containing five spectral features labeled A_1_, B_1_, C_1_, D_1_, and E_1_. The pre‐edge spectral features A_1_ and B_1_ in Fe‐based oxides are usually associated with the transitions from O 1s orbital to the hybridized orbitals of O 2p and Fe 3d.^[^ ^20^, ^21^ ^]^ Due to crystal field effects, the Fe 3d levels split into t_2g_ orbitals (xy, xz, and yz) and e_g_ orbitals (x^2^−y^2^, z^2^), giving rise to two contributions, which are labeled A_1_ and B_1_, respectively.^[^ ^22^ ^]^ The intensities of these spectral features corresponding to the states depend on the spin states and number of electrons in d‐orbitals.^[^ ^23^ ^]^ The comparable t_2g_: e_g_ intensity ratio of ZFO‐MSNi and ZFO (listed in Table S3, Supporting Information) indicate that the same high spin Fe^3+^ states are present in the two samples. While the lower intensities of A_1_ and B_1_ for ZFO‐MSNi compared to ZFO demonstrate that the transitions from O 1s orbital to the O 2p‐Fe 3d hybridized orbitals are slightly declined after the molten‐salt‐mediated introduction of Ni^2+^, indicating more occupation of O 2p‐Fe 3d hybridized orbitals in ZFO‐MSNi. This is likely due to the newly generated hybridization between Ni 3d and O 2p orbitals. In other words, the introduced Ni^2+^ weakens the hybridization between Fe 3d and O 2p states, which means the covalency of the Fe‐O bond in ZFO‐MSNi is weaker than that in ZFO. Figure 2c shows that the Fe L‐edge XANES spectra consist of two sets of peaks (L_3_ and L_2_), which correspond to electronic transitions from the 2p_3/2_ and 2p_1/2_ orbitals to the 3d excited states of the Fe atoms, respectively. As with the splitting of the Fe 3d orbitals into t_2g_ and e_g_ states, each Fe L‐edge peak consists of two contributions with a small pre‐peak followed by a main peak (labeled as A_2_, B_2_, and C_2_, D_2_, respectively). These spectral features also reveal that the high spin Fe^3+^ state remains after the introduction of Ni species in ZFO‐MSNi.^[^ ^24^ ^]^ Analogous to the O K‐edge spectra, the decreased spectral intensity of Fe L‐edge for ZFO‐MSNi relative to ZFO indicates more occupied Fe 3d states in ZFO‐MSNi,^[^ ^25^ ^]^ which clearly illustrates the chemical interaction between ZFO and the introduced Ni^2+^ through the molten‐salt‐mediated strategy. We suppose that a charge transfer from Ni 3d states and/or O 2p states to Fe 3d states is present in this case due to the orbital hybridization. Likewise, the decrease in the metal character of the high‐spin Fe^3+^ reveals a slightly weaker covalency of Fe‐O bonds after the introduction of Ni species in ZFO‐MSNi.^[^ ^26^ ^]^ Both the O K‐edge and Fe L‐edge XANES spectra clearly corroborate the existence of Ni—O interaction and its corresponding weakening effects on the covalency of Fe—O bonds in ZFO‐MSNi, which may significantly affect its PEC performance.

Moreover, as revealed in the X‐ray adsorption fine structure (XAFS) measurements, the Ni K‐edge near‐edge feature of ZFO‐MSNi resembles that of NiO and NiFe_2_O_4_ reference samples (Figure 2d), suggesting that the Ni species are positively charged as 2+. This is consistent with the above XPS and soft X‐ray absorption spectroscopy results. Fourier‐transformed k^3^‐weighted extended X‐ray absorption fine structure (EXAFS) in R space (Figure 2e) was further conducted to elucidate the coordination environment of Ni atoms. The absence of a peak at ≈ 2.4 Å which is attributed to Ni—Ni coordination in Ni metal excludes the formation of Ni nanoparticles or clusters in ZFO‐MSNi. By comparison with NiO and NiFe_2_O_4_, the peak at ≈ 1.9 Å of ZFO‐MSNi could be attributed to the Ni—O coordination in the first shell, while the peak at ≈ 2.8 Å can be ascribed to octahedral‐sited Ni—O—Fe coordination in the second shell.

According to the aforementioned characterization and analysis, it can be tentatively concluded that the atomically dispersed Ni^2+^ were inserted in surface B_oh_ sites on ZFO through the facile eutectic molten‐salt‐mediated strategy. More importantly, without destroying the nanorod array‐like morphology and the bulk crystal structure, the additional Ni_Oh_—O interaction and the associated relatively weaker Fe_Oh_—O bonding in the form of edge‐sharing octahedral configuration were successfully constructed in ZFO‐MSNi (as demonstrated in Figure 2f). We deduce that these newly generated interactions between surficial Ni, Fe, and O sites created some edge‐sharing Ni^2+^O_6_ and Fe^(3‐*σ*)+^O_6_ octahedral blocks that can perform the water oxidation reaction uniquely at the ZFO‐MSNi photoanode/electrolyte interface.

### 2.2 Photoelectrochemical (PEC) Performance

**Figure 3:**
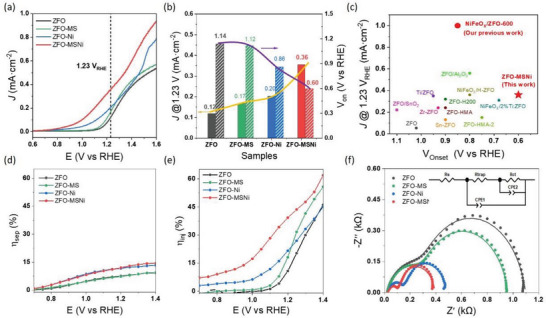
Photoelectrochemical measurement. a) *J*–*V* curves of samples. b) Extracted V_on_ and *J* @1.23 V_RHE_ from (a). c) Comparison of V_on_ and *J* @1.23 V_RHE_ in zinc ferrite dominant photoanodes. d) Charge separation efficiency *η*_sep_. e) Charge injection efficiency *η*_inj_. f) Nyquist plots of samples. (Solid line: fitting results).

Next, the PEC performance of ZFO‐MSNi and the control samples (ZFO, ZFO‐MS, ZFO‐Ni) were measured in 1.0 m NaOH electrolyte (pH = 13.6) under the standard 1‐sun irradiation (100 mW cm^−2^, simulated by 300 W Xe lamp facilitated with AM 1.5G filter) in the conventional three‐electrode system. As shown in the current density–voltage (*J*–*V*) curves (**Figure** **3**a,b), water oxidation commences at the photocurrent onset potential (V_onset_) of 0.60 V_RHE_ and 1.14 V_RHE_ for ZFO‐MSNi and ZFO, respectively. A remarkable cathodic shift of ≈ 450 mV relative to ZFO was achieved on ZFO‐MSNi, enabling its V_onset_ to reach a benchmark level compared to the zinc ferrite photoanodes from literature (displayed in Figure 3c, the corresponding details on PEC performance comparison are listed in Table S4, Supporting Information). Meanwhile, the photocurrent density of ZFO‐MSNi at 1.23 V_RHE_ is ≈ 0.36 mA·cm^−2^, which is three times that of ZFO. The control samples (ZFO‐MS and ZFO‐Ni) exhibited performance similar to the pristine ZFO, with only minor improvement, indicating obvious synergistic effects between the molten‐salt treatment and the octahedrally coordinated Ni^2+^ species. The incident photon‐to‐current efficiency (IPCE) values as a function of wavelength at the bias of 1.23 V_RHE_ for all photoanodes are plotted in Figure S4a (Supporting Information) (see Note S1 Supporting Information for calculation details). ZFO‐MSNi photoanode exhibits the highest IPCE values in the wavelength range from 360 to 550 nm, implying the most efficient utilization of solar energy. The maximum applied bias photon to current efficiency (ABPE) of 0.027% was achieved at 1.10 V_RHE_ for ZFO‐MSNi, > 10 times that for the pristine ZFO (Figure S4b, Supporting Information). Additionally, the stability of samples (Figure S4c, Supporting Information) was measured via chronoamperometry at 1.4 V_RHE_ under illumination. After the continuous test for 8 h, ZFO‐MSNi maintained > 93% of photocurrent, presenting quite good photochemical stability. During the long‐term stability measurement of the ZFO‐MSNi photoanode, the generated oxygen was collected and quantified by gas chromatography (Figure S4d, Supporting Information). The Faradaic efficiency is evaluated to be ≈ 96%, confirming that the sustained photocurrent of the ZFO‐MSNi photoanode was derived from a steady unity‐Faradaic‐efficiency oxygen evolution process. The small deviation can be attributed to two reasons: 1) There are other side reactions that may occur due to the contamination of electrolyte or electrode surface; 2) The generated oxygen is not fully detected as a small amount of gas may escape from the cell or dissolve in electrolyte. The retention morphology, crystal structure, surface elements composition, and electronic structure of ZFO‐MSNi photoanode (Figures S5 and S6, Supporting Information) after the long‐term stability test verified the good stability of ZFO‐MSNi as well. The improved PEC performance (especially for the remarkable cathodic shift) of ZFO‐MSNi strongly supports our inference that the structural variations on the ZFO surface by introducing B_oh_‐sited Ni^2+^ through the eutectic molten‐salt‐mediated strategy will promote the water oxidation reaction at the photoanode/electrolyte interface.

### 2.3 The Role of Molten‐Salt‐Mediated Ni^2+^ Insertion on ZFO Photoanodes

To reveal the underlying causes for the striking PEC water oxidation performance of ZFO‐MSNi, the photon absorption properties and the schematic band diagrams of samples were first checked by means of ultraviolet–visible diffuse reflectance spectra (UV–vis DRS) (Figure S7a, Supporting Information), valance band XPS spectrum (Figure S7b, Supporting Information). As depicted in Figure S7c (Supporting Information), the energy band structures of samples exhibit no significant changes, which is reasonable because the introduction of the small amount of Ni^2+^ only functions on the surface region in ZFO‐MSNi. These results exclude the light absorption contribution to the improved PEC performance of ZFO‐NSNi.

Then, using a sacrificial hole scavenger during PEC testing,^[^ ^27^ ^]^ the fundamental processes of photogenerated charge carrier transfer and separation under bias conditions were decoupled. The separation efficiency of photogenerated electron‐hole pairs in the bulk material (*η*_sep_) and the injection efficiency of holes in the surface region (*η*_inj_) for samples were evaluated in 1M  NaOH with the addition of 0.5 m H_2_O_2_ as a hole scavenger. The *η*_sep_ denotes the fraction of holes that reach the electrode/electrolyte interface without recombination in the bulk of the electrode, while *η*_inj_ represents the fraction of those holes at the interface that is successfully injected into the water oxidation. As described in Figure 3d,e (the corresponding *J*–*V* curves and calculation details are displayed in Figure S8 and Note S2, Supporting Information), ZFO‐MSNi possessed both the maximal *η*_sep_ and *η*_inj_. Moreover, the enhancement on *η*_inj_ compared to ZFO (from 4% to 34% at 1.15 V_RHE_ and from 0% to 12% at 0.9 V_RHE_) is much more significant than that on *η*_sep_ (from 7% to 11% at 1.15 V_RHE_ and from 3% to 6% at 0.9 V_RHE_), suggesting that it is the increased efficiency of hole injection at electrode/electrolyte interface that determines the striking PEC performance of ZFO‐MSNi. Additionally, when checking the values of *η*_sep_ and *η*_inj_ over the whole potential range (from 0.6 to 1.4 V_RHE_) for ZFO versus ZFO‐MS or ZFO‐Ni versus ZFO‐MSNi, the similar *η*_sep_ and obviously improved *η*_inj_ suggest that the molten‐salt treatment probably passivates some inherent surface states on ZFO without any alterations to the bulk material. Moreover, the improvements in both *η*_sep_ and *η*_inj_ when comparing ZFO‐Ni with ZFO show that the Ni^2+^ species play positive roles for both the charge carrier separation in bulk and the charge carriers injection to the water oxidation reaction at the interface.

Photoelectrochemical impedance spectroscopy (PEIS) also confirmed the above deductions. A representative two‐RC‐unit equivalent circuit was applied to fit the Nyquist plots of PEIS (the inset in Figure 3f, and the detailed fitting parameters are listed in Table S5, Supporting Information). As shown in Figure 3f, the radius of the semicircle curve for ZFO‐MSNi dramatically reduces compared with that of ZFO, indicating the decrease in both the resistance of charge transfer from bulk to surface states (*R*_trap_) and the resistance of charge injection from active sites to water oxidation reaction at electrode/electrolyte interface (*R*_ct_).^[^ ^28^, ^29^ ^]^ Quantitatively, the *R*_trap_ and *R*_ct_ values of ZFO‐MSNi photoanode are 107.7 Ω and 265.4 Ω, respectively. In contrast, the ZFO photoanode shows significantly larger *R*_trap_ and *R*_ct_ values of 401.1 Ω and 675.9 Ω, respectively. These are in line with the aforementioned improvement of *η*_sep_ and *η*_inj_. Likewise, the much larger *R*_ct_ for all samples compared to the corresponding *R*_trap_ also verifies that the hole injection to water at electrode/electrolyte interface is the determining step. Hereafter, we focus on the structural investigation at ZFO‐MSNi photoanode/electrolyte interface through additional advanced PEC techniques.

**Figure 4:**
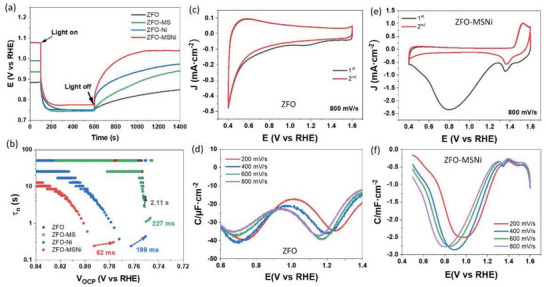
Identification of the roles of molten‐salt‐mediated introduction of Ni^2+^ on ZFO‐MSNi through PEC methods. a) The open circuit potential (OCP) transient decay profile. b) the OCP‐derived carrier lifetimes. c,d) Rapid‐scan cyclic voltammograms (RSVs) at 800 mV s^−1^ and the corresponding baseline‐corrected capacitance versus potential curves obtained from the RSV performed at 200, 400, 600, and 800 mV s^−1^ for ZFO, respectively. e,f) the counterparts for ZFO‐MSNi.

As known, both kinetic (decrease in overpotential, *η*) and thermodynamic (increase in photovoltage, V_ph_) factors can bring about a cathodic shift in PEC performance.^[^ ^30^ ^]^ We first conduct the open circuit potential (OCP) measurements in dark and under illumination for the ZFO as a function of the different treatments to electrochemically study their surface structural variations, since the steady‐state OCP measurements specifically track the thermodynamics without the influence of kinetic factors related to charge‐transfer.^[^ ^31^, ^32^ ^]^ As shown in **Figure** **4**a and Figure S9 (Supporting Information), the deviation from OCP_dark_ to 1.23 V_RHE_ for ZFO‐MSNi is minimal, suggesting that the Fermi level pinning which usually arises from the presence of surface states is significantly reduced after the molten‐salt‐mediated introduction of Ni^2+^.^[^ ^31^ ^]^ This result suggests that the molten‐salt‐mediated introduction of Ni^2+^ could heal some of the surface states on the pristine ZFO. Besides, the increased ∆OCP (ΔOCP = OCP_dark_−OCP_light_ = V_ph_) reveals the larger V_ph_ generated by the ZFO‐MSNi photoelectrodes, indicating an enhanced driving force of charge separation.^[^ ^32^ ^]^ Specifically, the value of V_ph_ for ZFO‐MSNi and ZFO is 0.3 V and 0.14 V, respectively. Therefore, we think the difference of 160 mV accounts for the corresponding thermodynamic contribution to the total cathodic shift of ≈ 450 mV.^[^ ^32^ ^]^

While for the residual cathodic shift of ≈ 290 mV, it can be attributed to the kinetic contributions (decrease in overpotential, *η*). As we revealed in the first section, additional Ni—O interaction and the corresponding relatively weaker Fe—O bonding were constructed in ZFO‐MSNi. It is likely that the newly introduced Ni^2+^ sites function as additional active sites for water oxidation, and the relatively weaker Fe—O bonding also gives rise to more active Fe^(3‐*σ*)+^ (0<*σ*<1) sites compared to Fe^3+^ sites on ZFO. These could bring about a reduced overpotential for the water oxidation reaction on the spinel photoanodes surface. To verify this speculation, we further evaluated the lifetime of the charge carriers as a function of OCP according to the following Equation:^[^ ^33^ ^]^

(1) $$\tau_{n}=\;-\frac{\kappa_{B}T}{e}{\left(\frac{\mathrm{dOCP}}{\mathrm{dt}}\right)}^{-1}$$

where *τ*_n_ is the carrier lifetime, *κ*_B_ is Boltzmann's constant, T is the temperature in K, e is the elementary charge, and dOCP/dt is the derivative of the OCP transient decay. The OCP transient decay evaluates the surface recombination between trapped electrons and reaction intermediates.^[^ ^34^ ^]^ In other words, the more rapid OCP transient decay indicates faster charge transfer from photoelectrode to water oxidation reaction. Figure 4b shows that the carrier lifetime of ZFO‐MSNi is 62 ms in the transient state, which is much smaller than that of the other three photoanodes (199 ms, 227 ms, and 2.11s for ZFO‐Ni, ZFO‐MS, and ZFO, respectively). The rapid decay kinetics suggests that ZFO‐MSNi possesses less charge trapping by surface states and faster charge injection to the water oxidation, which is in accord with our above PEC characterization and discussion.^[^ ^28^, ^35^ ^]^

Moreover, the rapid‐scan voltammetry (RSV) technique was further conducted to qualitatively evaluate the charge accumulation at the electrode/electrolyte interface. The validity of the RSV technique was established with ZFO and ZFO‐MSNi photoanodes by scanning at 200, 400, 600, and 800 mV s^−1^ (Figure 4c,d; Figure S10, Supporting Information). The extracted capacitance corresponding to the baseline‐corrected first rapid voltammetry scan at various speeds for ZFO and ZFO‐MSNi are displayed in Figure 4e,f, respectively. The broad capacitance peak in the range of 1.1–1.4 V_RHE_ for the pristine ZFO and that in the range of 0.5–1.2 V_RHE_ for ZFO‐MSNi can be attributed to the accumulation of holes on the water oxidation intermediates generated at the potential close to their V_onset_.^[^ ^36^, ^37^ ^]^ Obviously, the molten‐salt‐mediated introduction of Ni^2+^ provokes a remarkable increase in the capacitance, indicating more holes can participate in water oxidation. More importantly, we note that the capacitance peak centers are at ≈ 0.97 and ≈ 1.25 V at a scan speed of 200 mV s^−1^ for ZFO‐MSNi and ZFO, respectively. The observed cathodic shift of 280 mV, in this case, is quite close to the aforementioned residual cathodic shift of ≈ 290 mV when the thermodynamic contribution (increase in V_ph_) is considered singly for the total cathodic shift (≈ 450 mV) in ZFO‐MSNi.

**Figure 5:**
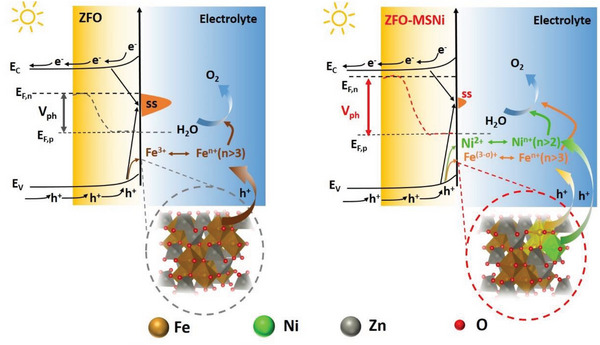
Schematic illustration of the energy band structure at ZFO electrolyte interface (left) and ZFO‐MSNi/electrolyte interface (right).

As demonstrated in **Figure** **5**, these are reasonable since the newly introduced Ni^2+^ united with the associated neighboring Fe^(3‐*σ*)+^ (0<*σ*<1) (in the weaker Fe—O bonging blocks) functioning as dual active sites on ZFO‐MSNi are more conducive to accept photogenerated holes compared to the pristine Fe^3+^ single sites on ZFO, and thus more easily participating in the water oxidation reaction. Additionally, ZFO‐MSNi possesses a decreased accumulation of holes at the semiconductor liquid junction due to fewer surface trapping states, which also reduces the kinetic bottleneck. All of the above experimental evidence supports the conclusion that the molten‐salt‐mediated introduction of Ni^2+^ forming Ni^2+^—Fe^(3‐*σ*)+^ dual sites in an edge‐sharing octahedral configuration effectively promotes the surface active sites of ZFO‐MSNi photoanode, resulting in both increased V_ph_ (thermodynamic contribution) and decreased *η* (kinetic contribution), thus enabling the significant cathodic shift on the V_onset_ for PEC water oxidation. As for the improved photocurrent, it is reasonable because the significantly boosted water oxidation reaction at electrode/electrolyte interface can drive and utilize the photogenerated carrier transfer from the bulk materials more powerfully and more efficiently. This can be affirmed by the increased *η*_sep_ as discussed at the beginning of this section. Nevertheless, further in‐situ/operando investigation as well as theoretical simulation of PEC water oxidation reaction at ZFO‐MSNi photoanode/electrolyte interface are needed for a deeper understanding of the effects of the constructed edge‐sharing octahedrally coordinated Ni—Fe dual active sites on its PEC performance.

## 3 Conclusion

In summary, a versatile eutectic molten‐salt mediated strategy for the introduction of Ni^2+^ is first reported to promote the active sites on spinel zinc ferrite photoanodes. The comprehensive structural characterization and PEC investigation reveal that the introduced octahedrally coordinated Ni^2+^ sites unite with the associated Fe^(3‐*σ*)+^ (0<*σ*<1) sites in edge‐sharing octahedral configuration significantly increase the photovoltage and reduce the overpotential for PEC water oxidation at ZFO‐MSNi photoanode/electrolyte interface. The simultaneously improved thermodynamic and kinetic processes are identified to be the underlying causes for the remarkable cathodic shift of ≈ 450 mV for the onset of the solar‐driven OER. This work provides new route to construct a highly catalytically active surface without destroying bulk structure for photoelectrodes on transparent conductive substrates as well as presenting deep insights by identifying the intrinsic factors at the photoelectrode/electrolyte interface that determine the performance, both of these can aid in advancing the field of solar‐driven PEC energy conversion.

## 4 Experimental Section

### Synthesis of ZFO‐MSNi Photoanodes

The ZFO‐MSNi photoanodes were synthesized by introducing Ni species to the surface of bare ZnFe_2_O_4_ photoanodes (labeled as ZFO) through a modified molten‐salt‐mediated strategy. Typically, bare ZFO photoanodes were prepared in the first step. The synthesis procedure of bare ZFO photoanodes was similar to that we have previously reported.^[^ ^15^ ^]^ First, *β*‐FeOOH nanorod‐array templates were fabricated on precleaned FTO substrates through a chemical bath deposition (CBD) method at 100°C for 6 h. The precursor for CBD consists of 0.15 m FeCl_3_^*^6H_2_O (99%, Sinopharm) and 1 m NaNO_3_ (99%, Sinopharm). Then excess zinc nitrate Zn(NO_3_)_2_^*^6H_2_O (99%, Sinopharm) aqueous solution was dropped on the obtained *β*‐FeOOH templates on a hot plate at 100°C. Subsequently, the samples were transferred to a tubular furnace at 600°C and kept for 20 min. Followed by natural cooling, the as‐obtained samples were soaked in concentrated NaOH (96%, Sinopharm) solution to remove ZnO on the surface due to the oxidation of the excess Zn(NO_3_)_2_. After final hydrogenation at 200°C for 1 h, bare ZFO photoanodes were obtained. Second, the bare ZFO photoanode was soaked in 5 mL 0.86 m LiNO_3_ (99%, Aladdin) and 1.14 m KNO_3_ (99%, Sinopharm) mixed molten salt aqueous solution for 3 h, in which 8 mg of NiCl_2_·6H_2_O (98%, Sinopharm) was pre‐dissolved in it. After drying them on a hot plate at 100 °C, the photoanodes were transferred to a tube furnace at 150 °C for 10 min under Ar atmosphere. Followed by cooling, washing, and drying the as‐obtained photoanodes were subjected to final hydrogenation at 200 °C for 10 min. Here, the target ZFO‐MSNi photoanodes were prepared. For comparison, control samples were also prepared from molten salts without Ni (labeled as ZFO‐MS), and using a conventional Ni impregnation method^[^ ^38^ ^]^ without molten salts (labeled as ZFO‐Ni).

### Materials Characterization

X‐ray diffraction (XRD) analysis was carried out on a high‐resolution X‐ray diffractometer for thin films (X'Pert3 MRD, Malvern Panalytical) with a Cu target (Cu K_*α*_ = 0.15418 nm), and the scanning rate was set as 10 ° min^−1^, which the 2*θ* angle scanning range was 10–70°. The morphology of all samples was characterized by transmission electron microscopy (TEM, JEM‐2011F) and scanning electron microscope (Genimi SEM 500). The high‐resolution transmission electron microscopy (HRTEM) and high‐angle annular dark‐field scanning transmission electron microscopy (HAADF‐STEM) images and the energy dispersive spectroscopy (EDS) mapping images were carried out on a JEOL JEM‐ARF200F atomic resolution analytical microscope. UV–vis DRS was tested by a UV–vis absorption spectrophotometer (Shimadzu UV‐2600, Japan) in the wavelength range of 300–700 nm, using BaSO_4_ as the reference. The X‐ray photoelectron spectroscopy (XPS) measurements were performed on an X‐ray photoelectron spectrometer (VG ESCALAB MK II) with Mg K_*α*_ = 1253.6 eV as the exciting source. The content of Ni was determined by inductively coupled plasma‐atomic emission spectrometry (ICP‐AES) (VG, USA). The soft X‐ray absorption spectroscopy at Ni L‐edge, Fe L‐edge, and O K‐edge were measured in total electron yield (TEY) mode at the BL12B‐*α* beamline of National Synchrotron Radiation Laboratory (NSRL, Hefei). The X‐ray absorption fine structure (XAFS) measurements at Ni K‐edge of samples were carried out in fluorescence mode at the 1W1B station in Beijing Synchrotron Radiation Facility (BSRF, Beijing). The standard Ni foil was supplied by 1W1B station at BSRL, and the NiO (99.99%, powder) standard sample was purchased from Aladdin Reagent (Shanghai) Co., Ltd.

### Photoelectrochemical (PEC) Measurements

PEC measurements were measured by using a standard three‐electrode system (samples were used as the working electrode; Ag/AgCl electrode were used as the reference electrode; Pt electrodes were used as the counter electrode) with an electrochemical workstation (CHI660, CH Instruments, Inc.) under 100 mW·cm^−2^ AM 1.5G simulated sunlight (1‐sun, CEL–HXF300–T3 Xe lamp, CEAULIGHT, China). All the measured potentials versus Ag/AgCl reference electrode were converted to the potentials versus reversible hydrogen electrode (RHE) by the Nernst equation: *E*_RHE_ = $E_{\mathrm{Ag}/\mathrm{AgCl}}$ + 0.059 pH + $E_{\mathrm{Ag}/\mathrm{AgCl}}^{\theta }$ ($E_{\mathrm{Ag}/\mathrm{AgCl}}^{\theta }$ = 0.1976 at 25°C). The current–potential (*J*−*V*) curves of the samples were measured by linear sweep voltammetry using 1 m NaOH (pH = 13.6) as electrolyte at the potential range from −0.5 to 0.6 V_Ag/AgCl_, and the scan rate was 50 mV s^−1^, the sensitivity was 10^−3^ A V^−1^. The long‐term stability measurements were conducted at 1.4 V versus RHE under 1‐sun illumination using chronoamperometry method. To evaluate Faradic efficiency of the photoanode, the sealed photoelectrochemical cell was pre‐purged by Ar for 1 h to remove O_2_ in the system and the O_2_ evolution during the long‐term stability measurements was collected every hour by gas chromatography (GC7920, CEAULIGHT, China) with a thermal conductivity detector, 5 Å molecular sieve columns, and argon carrier gas. The photoelectrochemical impedance spectra (PEIS) of the samples were obtained by A.C. impedance method, under 1 sun conditions in an electrolyte of 1 m NaOH, at the potential of 1.23 V_RHE_ and the frequency range from 0.01 to 10^6^ Hz. The open‐circuit potential was measured by the open‐circuit potential‐time method, with the 1 m NaOH which was saturated with oxygen as the electrolyte. Rapid‐scan cyclic voltammograms (RSVs) were measured by a two‐step process with a minimum delay in between them. First, the electrode was held at 1.8 V_RHE_ under constant illumination for 60 s recording the photocurrent (chronoamperometry) followed by the interruption of the light and recording the cyclic voltammograms (starting potential 1.6 V_RHE_ and scanning in the negative direction).

## Conflict of Interest

The authors declare no conflict of interest.

## Supporting information

Supporting Information

## Data Availability

The data that support the findings of this study are available in the supplementary material of this article.
