# Supplementary material for: Edge‐Sharing Octahedrally Coordinated Ni—Fe Dual Active Sites on ZnFe2O4 for Photoelectrochemical Water Oxidation
Source: Adv Sci (Weinh). 2023 Jun 1;10(22):2301869. doi: 10.1002/advs.202301869 (PMC10401156; doi:10.1002/advs.202301869)
Supplement: Supplementary file 1 — Supporting Information [file ADVS-10-2301869-s001.pdf]

ADVANCED SCIENCE
Open Access

Supporting Information

for *Adv. Sci.*, DOI 10.1002/advs.202301869

Edge-Sharing Octahedrally Coordinated Ni–Fe Dual Active Sites on $ZnFe_2O_4$ for Photoelectrochemical Water Oxidation

Zhiyong Jiang, Xiaodi Zhu, Zhiyu Wang, Wei Liu, Wensheng Yan, Kevin Sivula* and Jun Bao**

Supporting Information

Edge-sharing Octahedrally Coordinated Ni-Fe Dual Active Sites on $ZnFe_2O_4$ for Photoelectrochemical Water Oxidation

Zhiyong Jiang[a†], Xiaodi Zhu[a†]*, Zhiyu Wang[a], Wei Liu[d], Wensheng Yan[a], Kevin Sivula[e]*, Jun Bao[a,b,c]*

[a] National Synchrotron Radiation Laboratory, University of Science and Technology of China, Hefei, Anhui 230029, China

[b] Key Laboratory of Precision and Intelligent Chemistry, University of Science and Technology of China, Hefei, Anhui 230026, China

[c] iChEM (Collaborative Innovation Center of Chemistry for Energy Materials), Hefei, Anhui 230029, China

[d] State Key Laboratory of Fine Chemicals, School of Chemical Engineering, Dalian University of Technology, Dalian 116024, China

[e] Laboratory for Molecular Engineering of Optoelectronic Nanomaterials (LIMNO), École Polytechnique Fédérale de Lausanne, Station 6, 1015, Lausanne, Switzerland

*Corresponding author. E-mail:

zhuxiaodi@ustc.edu.cn; kevin.sivula@epfl.ch; baoj@ustc.edu.cn

[†]These authors contributed equally to this work.

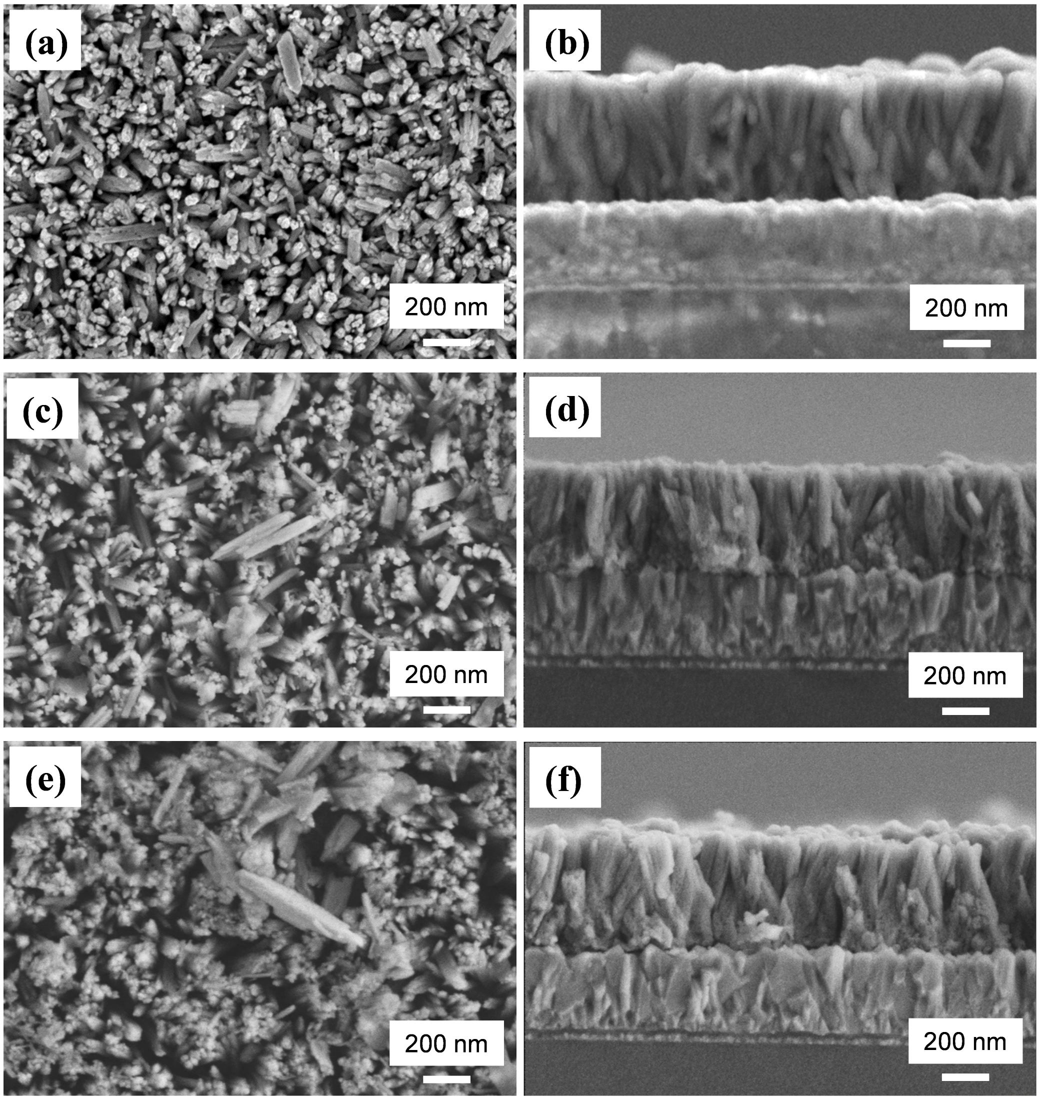

Figure S1. Top view (left) and cross-sectional (right) SEM images of different samples. (a)(b) ZFO, (c)(d) ZFO-MS, and (e)(f) ZFO-Ni.

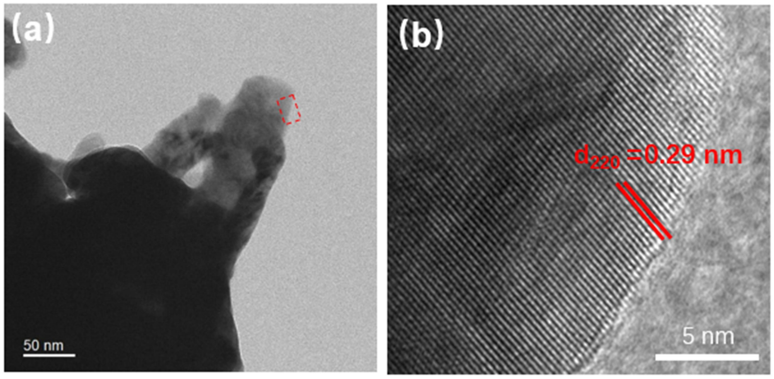

Figure S2. (a) TEM and (b) HTREM images of fresh ZFO-MSNi nanorods.

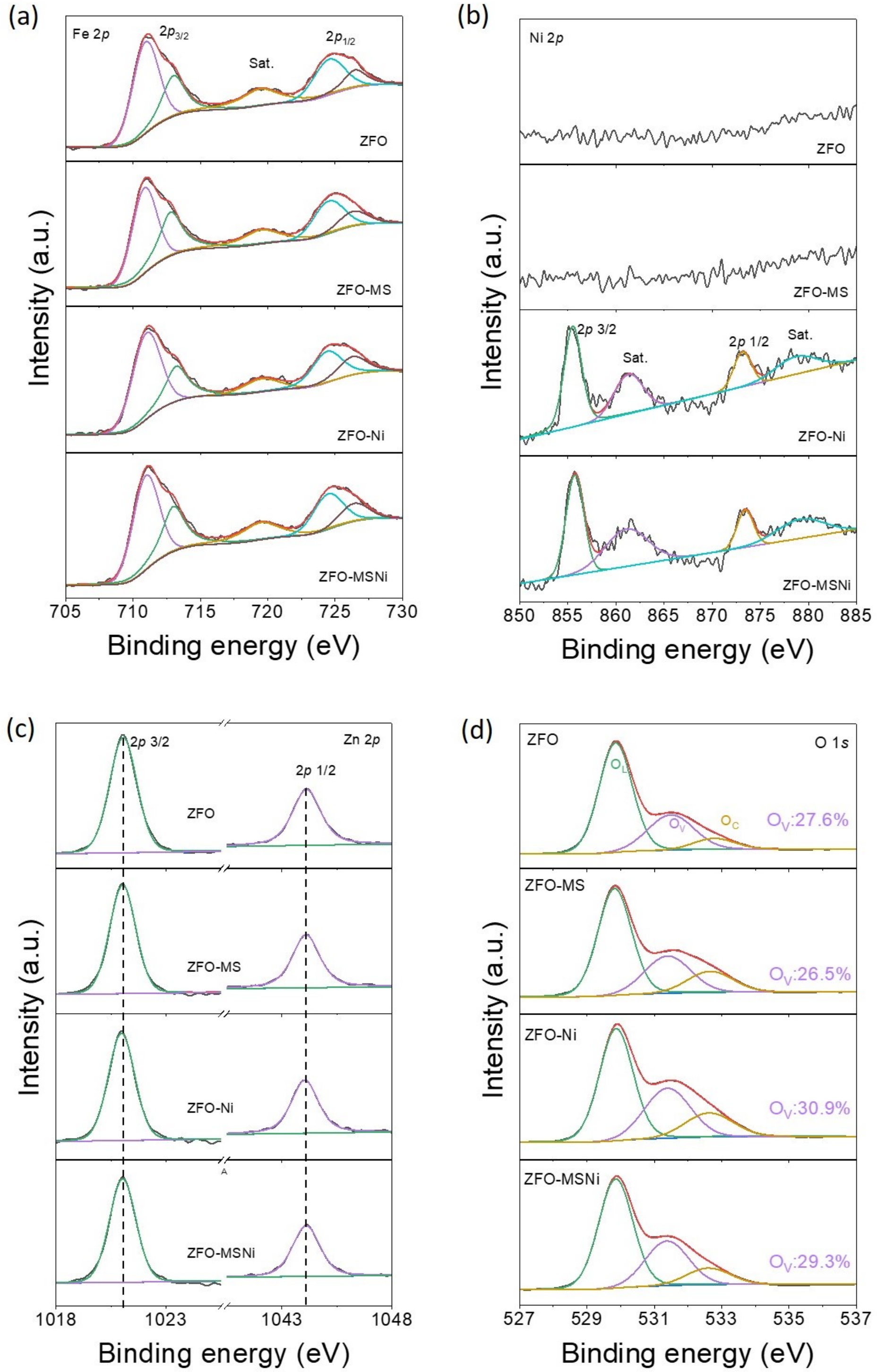

Figure S3. XPS spectra of samples. (a) Fe 2*p*, (b) Ni 2*p*, (c) Zn 2*p*, and (d) O 1*s*.

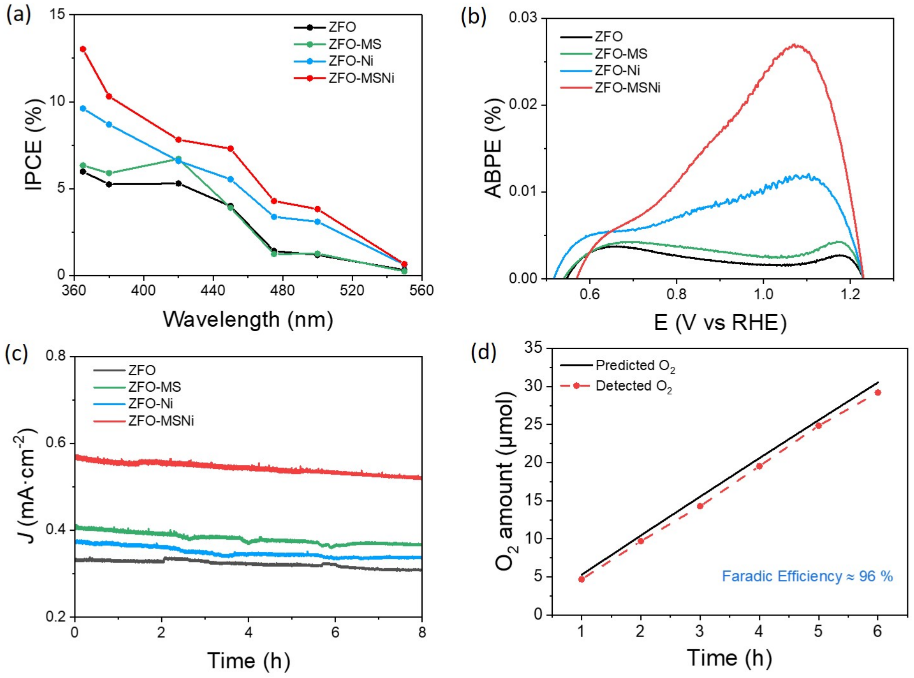

Figure S4. Photoelectrochemical performance of different samples. (a) IPCE, (b) ABPE, (c) i-t curves at 1.4 V_{RHE} under 1-Sun illumination, and (d) the O_2 gas evolution during the long-term stability measurements.

Note 1:

The incident photon-to-current conversion efficiency (IPCE) was determined by a Xe lamp (300 W, CEAULIGHT) with corresponding filters at 1.23 V_{RHE} in the same electrolyte (1 M NaOH). The IPCE values were calculated by the equation (S4-1):

$$IPCE = \frac{1240 \times J(mW \cdot cm^{-2})}{P_{light}(mW \cdot cm^{-2}) \times \lambda(nm)} \# (S4-1)$$

where J is the measured photocurrent density, P_{light} is the calibrated illumination power at the specific wavelength, and λ is the corresponding wavelength of the incident light.

The applied bias photon to current efficiency (ABPE) was calculated by the equation (S4-2):

$$ABPE = \frac{J(mW \cdot cm^{-2}) \times (1.23 - E)}{P_{irradiation}} \# (S4-2)$$

where J is the photocurrent density, E is applied bias potentials, $P_{irradiation}$ is the calibrated illumination power.

Faraday efficiency is calculated by collecting O_2 gas during the stability test. The theoretical oxygen generation amount was calculated by the equation (S4-3):

$$the\ amount\ of\ predicted\ O_{2\,predicted} = \frac{It}{nF} \#(S4-3)$$

Where $O_{2\,predicted}$ represents the theoretical oxygen generation amount; I represents the current density at 1.4 V_{RHE}; t represents the time; n represents the number of electron transfers for OER reaction, and the F represents the Faraday's constant (96500 $C{\cdot}mol^{-1}$). The Faraday efficiencyη_{FE} is calculated by the equation (S4-4):

$$\eta_{FE} = \frac{the\ amount\ of\ O_{2\,detected}}{the\ amount\ of\ O_{2\,predicted}} * 100\% \qquad (S4-4)$$

Where $O_{2\,detected}$ represents the detected oxygen generation amount under stability test.

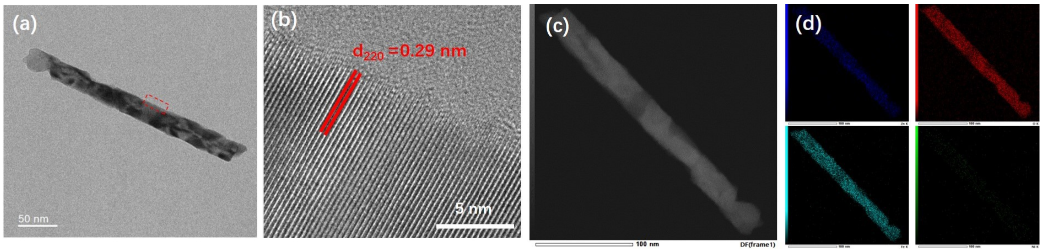

Figure S5. (a) TEM, (b) HTREM,(c) STEM images and corresponding EDS mapping of used ZFO-MSNi photoanode after long-term stability measurement.

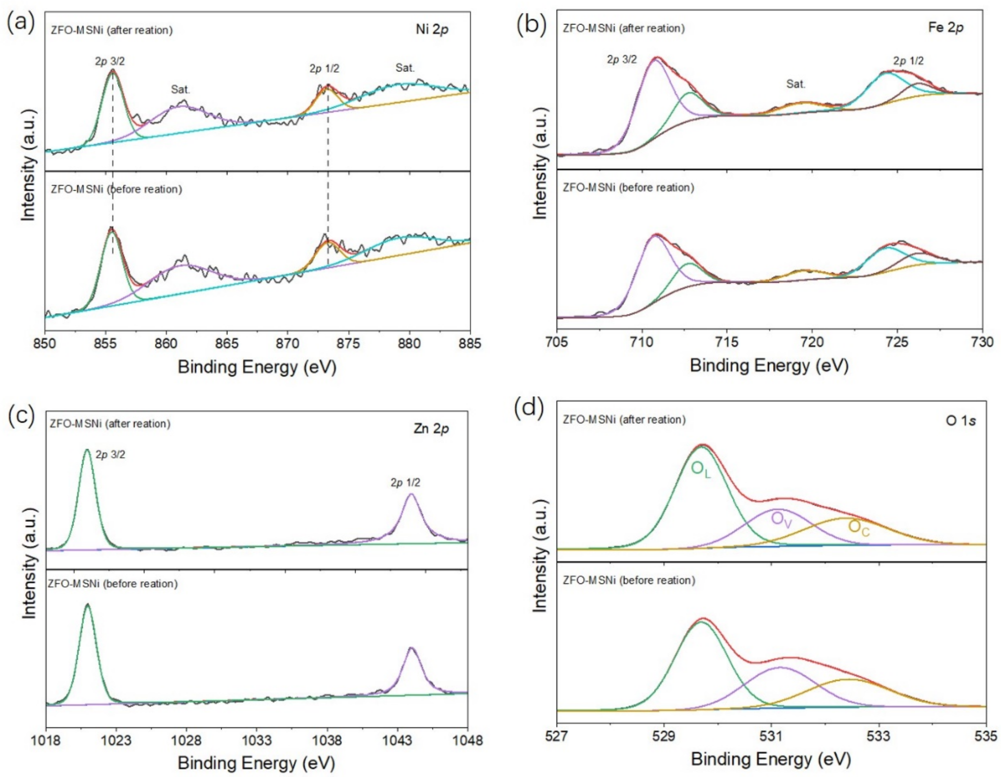

Figure S6. (a) Ni 2p, (b) Fe 2p, (c) Zn 2p and (d) O 1s High resolution XPS of ZFO-MSNi before and after long-term stability test.

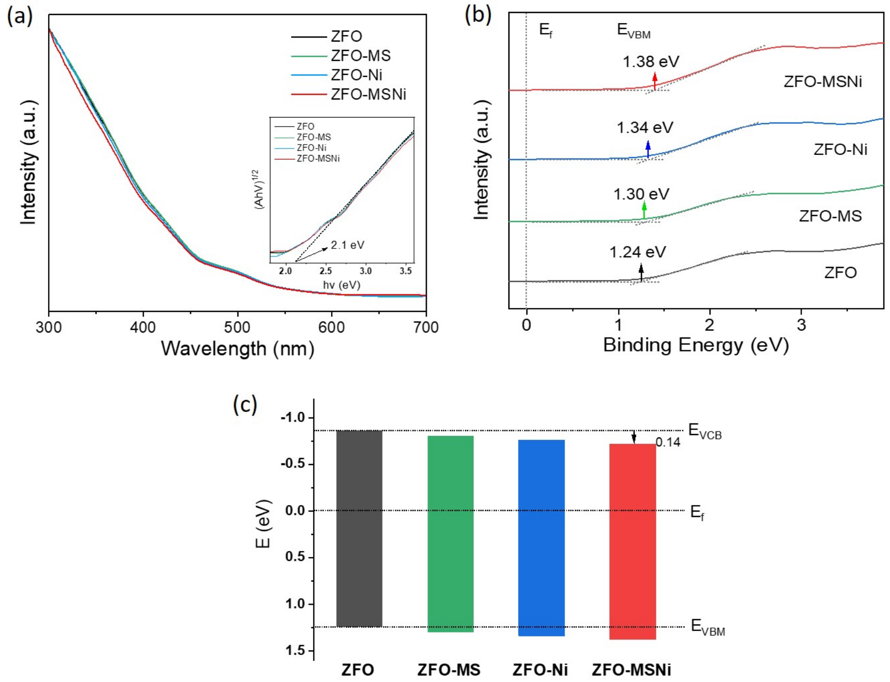

Figure S7. (a) UV-Vis DRS, (b) XPS valence band spectrum, (c) Energy band structure.

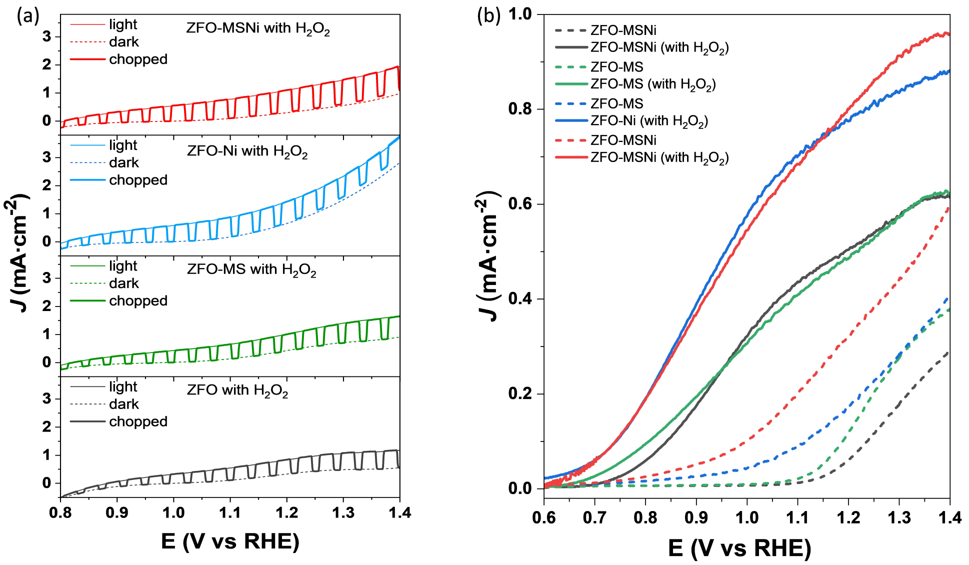

Figure S8. (a) *J*-V curves and chopped *J*-V curves with H_2O_2, and (b) *J*-V curves with and without H_2O_2.

Note 2:

The photocurrent density under water oxidation conditions was calculated by the equation (S8–1):

$$J_{H_2O} = J_{abs} \times \eta_{sep} \times \eta_{inj} \#(S8-1)$$

where J_{abs} represents the theoretical maximum photocurrent density. The value of J_{abs} for ZFO is 6.55 mA·cm^{-2}, which was calculated by the overlapped area integration of standard solar absorption spectrum and UV-Vis absorption spectrum. The η_{sep} (related to the charge separation efficiency of photogeneration hole which transfers to the surface) and η_{inj} (related to the charge injection efficiency of holes on the surface that inject into the electrolyte) could be determined using the following formulas (S8-2) and (S8-3)：

$$\eta_{sep} = \frac{J_{H_2O_2}}{J_{abs}} \times 100\% \#(S8-2)$$

$$\eta_{inj} = \frac{J_{H_2O}}{J_{H_2O_2}} \times 100\% \#(S8-3)$$

where $J_{H_2O_2}$ and J_{H_2O} are the photocurrent density in the electrolyte with and without hole scavenger H_2O_2, respectively. When hole scavenger H_2O_2 was added to the electrolyte, assuming the charge injection efficiency is 100%.

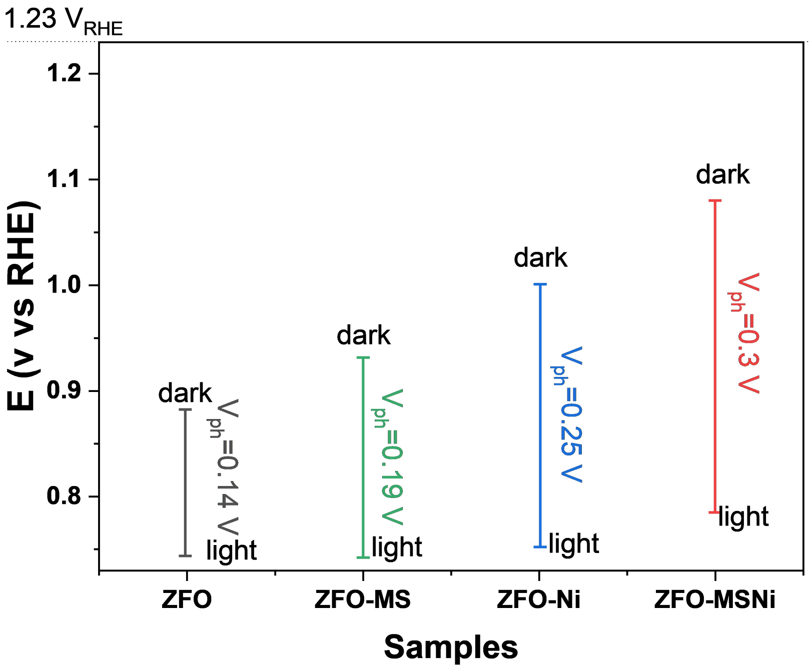

Figure S9. OCP values of different photoanodes.

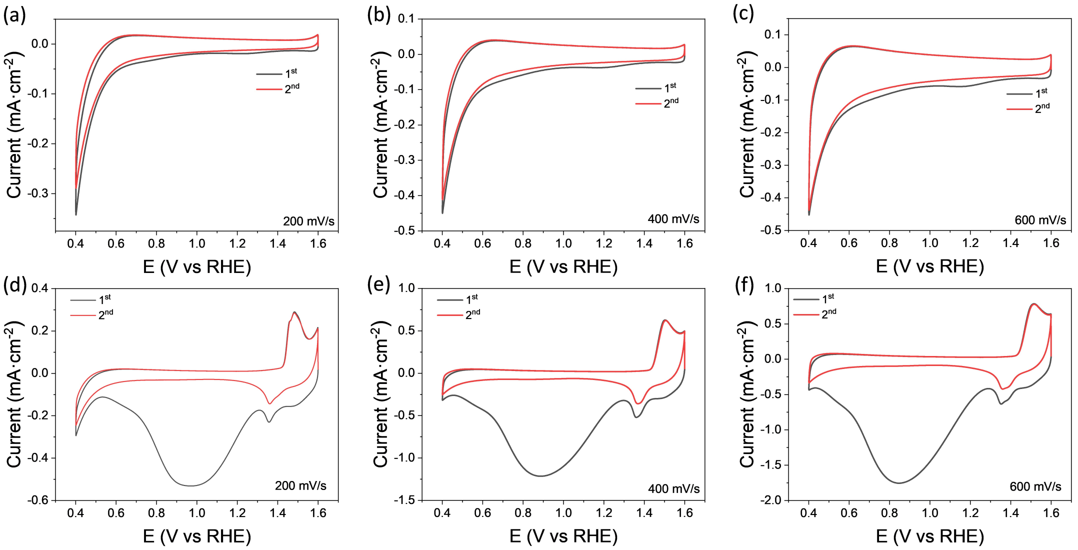

Figure S10. Rapid-scan cyclic voltammograms (RSVs) as a function of the scan rate (200-600 mV/s) for the ZFO (a)(b)(c), and the ZFO–MSNi (d) (e)(f), respectively.

Table S1. Elements composition in surface region evaluated from XPS spectra.

	ZFO		ZFO-MS		ZFO-Ni		ZFO-MSNi	
	**Atomic conc. %**	**Mass conc. %**	**Atomic conc. %**	**Mass conc. %**	**Atomic conc. %**	**Mass conc. %**	**Atomic conc. %**	**Mass conc. %**
Fe	11.9	31.3	8.99	26.2	7.3	21.5	9.83	25.9
Zn	4.62	14.1	3.24	11	2.61	8.9	3.56	10.9
Ni	0	0	0	0	1.79	5.6	3.05	8.5
C	43.3	24.4	49.79	31.1	48.79	30.8	43.14	24.3
O	40.18	30.2	37.98	31.7	39.51	33.2	40.43	30.4

Table S2. Elements composition of ZFO-Ni and ZFO-MSNi determined by ICP-OES.

	**μg/mL**		**Mass conc. %**		**μmol**	
	**ZFO-Ni**	**ZFO-MSNi**	**ZFO-Ni**	**ZFO-MSNi**	**ZFO-Ni**	**ZFO-MSNi**
Fe	33.989	31.595	21.24	22.56	3.043	2.829
Zn	18.499	17.469	11.56	12.48	1.415	1.336
Fe：Zn	-	-	-	-	2.15:1	2.12:1
Ni	0.580	0.657	0.36	0.47	0.049	0.056

Table S3. Intensity ratio of t_{2g} : e_g from O k-edge in Figure 2b

	**ZFO**	**ZFO-MSNi**
t_{2g}	1.15	1.21
e_g	1.23	1.29
t_{2g} : e_g	0.93	0.93

Table S4. Photoelectrochemical water oxidation performance of $ZnFe_2O_4$ photoanodes used as a single light absorber.

Sample	Synthesis method	Co-catalyst	Onset potential [V_{RHE}]	*J* at 1.23 V_{RHE} [mA/cm^2]
$ZnFe_2O_4$[S1]	AACVD	none	0.82	0.35
ZFO2[S2]	Electrospray	none	1.02	0.053
m-$ZnFe_2O_4$[S3]	ALD with inverse opal structure	none	0.90	0.26
ZFO/SnO_2(helix)[S4]	Conversion of FeOOH with Zn solution followed by CTA	none	1.1	0.22
ZFO/Al_2O_3[S5]	Conversion of FeOOH with Zn solution followed by CTA and H_2 treatment and ALD	none	0.85	0.35
ZF550-HMA[S6]	Conversion of FeOOH with Zn solution followed by CTA and HMA	none	0.9	0.24
ZFO-H200[S7]	Conversion of FeOOH with Zn solution followed by CTA and H_2 treatment	none	0.83	0.32
ZF-HMA-2[S8]	Conversion of FeOOH with Zn solution followed by HMA	none	0.75	0.15
H-ZFO[S9]	Conversion of FeOOH with Zn solution followed by CTA and H_2 treatment	$NiFeO_x$	0.8	0.36
ZFO-600[S1] (Previous work)	Conversion of FeOOH with Zn solution followed by CTA and H_2 treatment	$NiFeO_x$	0.85	1.00
2%Ti:ZF_{800}[S10]	Conversion of FeOOH with Zn solution followed by CTA and Ti doping	$NiFeO_x$	0.68	0.31
ZFO3[S11]	Conversion of FeOOH with Zn solution followed by CTA and Sn doping	none	0.9	0.13
Zr-ZFO-15[S12]	Conversion of FeOOH with Zn solution followed by	none	0.7	0.24

	CTA and doping			
Ti(6%)$ZnFe_2O_4$[S13]	Spray pyrolysis and Ti doping	none	0.95	0.56
ZFO-MSNi (This work)	Conversion of FeOOH with Zn solution followed by CTA and MS	none	0.64	0.36

Note: Under 1 Sun conditions in an electrolyte of 1M NaOH. AACVD (aerogel assisted chemical vapor deposition), CTA (conventional thermal annealing), HMA (hybrid microwave annealing), ALD (atomic layer deposition), Substrate: FTO.

Table S5. The fitting results obtained from EIS spectra.

	R_s/Ω cm^2	R_{trap}/Ω cm^2	CPE1/F	R_{ct}/Ω cm^2	CPE2/F
**ZFO**	21.23	401.1	$6.74*10^{-5}$	675.9	$2.46*10^{-4}$
**ZFO-MS**	21.52	353.1	$5.40*10^{-5}$	584.6	$3.13*10^{-4}$
**ZFO-Ni**	21.79	162.8	$2.31*10^{-4}$	298.6	$2.38*10^{-3}$
**ZFO-MSNi**	21.94	107.7	$3.43*10^{-4}$	265.4	$3.04*10^{-3}$

Reference:

[S1] A. A. Tahir, K. G. U. Wijayantha, *J. Photochem. Photobiol. A.* **2010**, 216, 119-125.

[S2] M. Wang, Y. Sun, H. Chen, Y. Zhang, X. Wu, K. Huang, S. Feng, *CrystEngComm* **2017**, 19, 772.

[S3] A. G. Hufnagel, K. Peters, A. Müller, C. Scheu, D. Fattakhova-Rohlfing, T. Bein, *Adv. Funct. Mater.* **2016**, 26, 4435.

[S4] J. H. Kim, I. Y. Choi, J. H. Kim, J. Kim, Y. K. Kim, J. K. Kim, J. S. Lee, *Small* **2021**, 17, 2103861.

[S5] Y. Advanced Functional MaterialsJournal of Materials ResearchLiu, M. Xia, L. Yao, M. Mensi, D. Ren, M. Grätzel, K. Sivula, N. Guijarro, *Adv. Funct. Mater.* **2021**, 31, 2010081.

[S6] J. H. Kim, J. H. Kim, J.-W. Jang, J. Y. Kim, S. H. Choi, G. Magesh, J. Lee, J. S. Lee, *Adv. Energy Mater.* **2015**, 5.

[S7] J. H. Kim, Y. J. Jang, J. H. Kim, J. W. Jang, S. H. Choi, J. S. Lee, *Nanoscale* **2015**, 7, 19144.

[S8] J. H. Kim, Y. J. Jang, S. H. Choi, B. J. Lee, M. H. Lee, J. S. Lee, *ACS Sustain. Chem. Eng.* **2018**, 7, 944.

[S9] N. Guijarro, P. Bornoz, M. Prévot, X. Yu, X. Zhu, M. Johnson, X. Jeanbourquin, F. Le Formal, K. Sivula, *Sustainable Energy Fuels* **2018**, 2, 103.

[S10] J. H. Kim, J. H. Kim, J. H. Kim, Y. K. Kim, J. S. Lee, *Sol. RRL* **2019**, 4, 1900328.

[S11] J. W. Park, M. A. Mahadik, G. W. An, S. Y. Lee, G. Piao, S. H. Choi, W.-S. Chae, H.-S. Chung, H. Park, J. S. Jang, *Solar Energy Materials and Solar Cells* **2018**, 187, 207.

[S12] S. Kim, M. A. Mahadik, W.-S. Chae, J. Ryu, S. H. Choi, J. S. Jang, *Appl. Surf. Sci.* **2020**, 513, 145528.

[S13] Y. Guo, N. Zhang, X. Wang, Q. Qian, S. Zhang, Z. Li, Z. Zou, *J. Mater. Chem. A* **2017**, 5, 7571.
